# The type 1 and type 3 immune responses underlying the tissue inflammation associated with NOX2 deficiency

**DOI:** 10.3389/fimmu.2026.1848916

**Published:** 2026-07-16

**Authors:** Wan-Ting Cheng, Miao-Shan Lin, Tzu‐Yi Chan, Chun-Hsin Wu, Chi-Chang Shieh

**Affiliations:** 1 Institute of Clinical Medicine, College of Medicine, National Cheng Kung University, Tainan, Taiwan; 2 Department of Internal Medicine, National Cheng Kung University Hospital, College of Medicine, Division of Allergy, Immunology and Rheumatology, National Cheng Kung University, Tainan, Taiwan; 3 Department of Pediatrics, National Cheng Kung University Hospital, Tainan, Taiwan

**Keywords:** Autoimmunity, CGD, Gene carrier, IL-1 β, NADPH oxidase2 (NOX2), NLRP3 inflammasome, Th1 inflammation, Th17 inflammation

## Abstract

Phagocytic nicotinamide adenine dinucleotide phosphate (NADPH) oxidase (NOX2) is the primary source of reactive oxygen species (ROS) in leukocytes for host defense and immune signaling. Loss-of-function mutations in NOX2 leads to chronic granulomatous disease, a life-threatening immunodeficient disorder with defective phagocyte function to produce ROS for killing bacteria and fungi. NOX2, however, is not restricted to phagocytic leukocytes; it is also present in various immune cell types, with expression levels varying among them. Disrupted redox balance in CGD patients predisposes them to develop Th1/Th17-type (type 1 and type 3) inflammation leading to severe tissue inflammation in organs including joints, lungs, heart and kidneys. The high Th1 cytokines in tissues induce macrophage and T cell activation, which makes a positive feedback loop to recruit more immune cells to facilitate the granuloma formation in CGD patients. Moreover, IFN-γ has been shown to play a critical role in age-associated B cells (ABC) differentiation by inducing T-bet expression through STAT1 activation in B cells and elevated autoantibody production. The data suggested that the IFN-γ-rich Th1 environment resulting from NOX2 deficiency may promote ABC differentiation via the IFN-STAT axis. Previous studies showed that CGD patients and NOX2 deficient mice have stronger Th17 type inflammation and higher type 3 effector cytokine production. Overexpression of IL-1β following NLRP3 inflammasome-dependent or -independent activation aggravates Th17 hyperinflammation in CGD patients, who are prone to develop comorbidities including pulmonary fibrosis, arthritis, lupus-like nephritis and increased risk of cardiovascular diseases. In this review, we summarize the pivotal role of NOX2 in the balance of redox homeostasis and its relationship with type 1/type 3 inflammation. The key issues to be explored on NOX2-deficiency-associated tissue inflammation include: (i) to elucidate the cellular and molecular bases for the Th1 and Th17 hyperinflammation in CGD-related comorbidities and how the inflammation evolves to autoimmunity (ii) how subjects with low ROS production (e.g. gene carriers of CGD mutations) are affected by these pathogenic tissue inflammation, and (iii) developing therapies that restore NOX2 function in targeted immune cells, enhancing ROS-driven microbial killing while preventing oxidant stress-induced tissue damage, and addressing autoinflammation and autoimmune issues in CGD patients.

## 1 Introduction

The dysfunction of nicotinamide adenine dinucleotide phosphate (NADPH) oxidase-2 (NOX2), resulting in an impaired respiratory burst and reduced production of reactive oxygen species (ROS) in leukocytes, was identified during the latter half of the twentieth century as the underlying cause of the inborn error of immunity (IEI) known as chronic granulomatous disease (1, 2). ROS are short-lived, highly reactive radical or non-radical oxygen-derived molecules that are essential for the microbiocidal activity of phagocytic leukocytes through interactions with diverse biological molecules. Cellular ROS originate from multiple sources, including NADPH oxidases (NOXs), mitochondria, endoplasmic reticulum (ER), and peroxisomes; however, most of these pathways generate ROS as metabolic by-products (1–4). In contrast, NADPH oxidases are specialized membrane-bound enzymes dedicated to ROS production. The NOX family comprises seven isoforms: NOX1–5 and dual oxidases DUOX1 and DUOX2 (1, 5, 6). It has been well known that redox regulation is critical for maintaining physiological homeostasis. While excessive ROS production is widely associated with inflammation and tissue damage, insufficient ROS levels can also result in pathological conditions, including chronic inflammation and autoimmunity (2, 4, 7–9). Accumulating evidence indicates that ROS, particularly hydrogen peroxide (H_2_O_2_), which is predominantly produced by NOXs, function as second messengers and participate in diverse cellular processes, including activation, differentiation, proliferation, polarization, survival, cytokine production, and migration (2, 4, 10–13). Emerging evidence suggests that the balance of intracellular ROS sources influences immune cell fate, shaping regulatory or inflammatory responses that contribute to NOX2-deficiency-associated inflammation and CGD comorbidities (7, 14–17). In this review, we discuss the cellular and molecular mechanisms underlying Th1- and IL-1β/Th17-driven hyperinflammation and its progression to autoimmunity in CGD. We further examine how these mechanisms could influence potential NOX2-targeting therapeutic strategies for treating autoinflammatory and autoimmune manifestations in CGD and other conditions associated with NOX2 deficiency.

### 1.1 NOX2 expression and function in immune cells

As both excessive and insufficient ROS levels can lead to diseases, intracellular ROS concentrations are tightly controlled by ROS production, antioxidants and scavenging systems under physiological conditions (2, 4, 18, 19). The biological effects of ROS depend on their cellular context, concentrations, duration of exposure, and subcellular origins (20). ROS function as signaling intermediates that regulate redox-sensitive proteins through post-translational modifications, including reversible or irreversible cysteine chemical alterations including S-glutathionylation, S-nitrosylation, and disulfide bond formation. ROS can oxidize cysteine residues in protein tyrosine phosphatases (PTPs), prolonging kinase activation and modulating signaling pathways that control transcription factors including NF-κB, JAK-STAT, and PPAR-γ (4, 7, 10–13). Through these mechanisms, ROS regulate inflammatory gene expression, cell proliferation, cell differentiation and trafficking, among other critical immune cell functions.

Among the NADPH oxidase family, NOX2 is predominantly expressed in immune cells and consists of the membrane-bound flavocytochrome b_558_ (gp91^phox^ and p22^phox^) and cytosolic components (p47^phox^, p67^phox^ and p40^phox^), together with the small GTPases Rac1 or Rac2. To avoid the excessive production of its main product, superoxide (O_2_^-^), and maintain redox homeostasis, NOX2 activity needs to be precisely regulated. In the inactive state, NOX2 components remain dissociated. Upon activation by signals transmitted through integrins, Fc receptors, C-type lectin receptors, or G-protein-coupled receptors, the cytosolic subunits become phosphorylated, translocate to the membrane, and assemble with flavocytochrome b_558_, thereby triggering superoxide production and the oxidative burst. In contrast, Toll-like receptors (TLRs) and cytokine receptors primarily provide priming signals that enhance molecular phosphorylation and prepare for subsequent NOX2 activation. NOX2-derived ROS are essential for host defense against bacteria and fungi by mediating microbial killing in phagolysosomes. Loss-of-function variants in gp91phox (*CYBB*), p22phox (*CYBA*), p47phox (*NCF1*), p67phox (*NCF2*), and p40phox (*NCF4*) are designated as the causes of phagocyte-related IEI, clinically presenting as CGD (21, 22).

The NOX2 complex is predominantly expressed in professional phagocytes, including neutrophils, monocytes, macrophages, and dendritic cells, where it coordinates with efficient pathogen engulfment, clearance, antigen processing and antigen presentation. The high expression of *CYBB*, *NCF2*, and *NCF4* in neutrophils underscores their central contribution to oxidative burst-mediated antimicrobial defense. On the other hand, lymphocyte populations, including T cells, NK cells, plasma cells, and B cells, exhibit markedly lower but still significant expression of most NOX2 subunits, particularly in B cells (7, 8, 23, 24). This distribution suggests that NOX2 may primarily function as a signaling regulator in adaptive immune cells, in contrast to its classical microbicidal role in phagocytes. In B cells, NOX2-derived ROS have been proposed to modulate phagosome pH and antigen proteolysis, thereby affecting peptide processing, antigen presentation, and interaction with T cells. Therefore, NOX2 deficiency may affect immune tolerance to self-antigens, promote aberrant B cell activation and increase susceptibility to autoimmunity (8, 25–27). (see section 2 for further discussion).

Although findings vary among different cell types, growing evidence indicates that NOX2-derived ROS regulate the tissue environment by promoting anti-inflammatory, non-autoreactive immune cell phenotypes, thereby reducing autoinflammation and autoimmunity (1, 7, 8, 28). The dual effects of NOX2-derived ROS likely depend on their cellular source, disease context and stage, ROS levels, and cell type-specific intrinsic and extrinsic influences within tissues. NOX2 hence may serve as a functional bridge between innate and adaptive immunity, with cell type-specific expression levels corresponding to distinct immunological functions.

### 1.2 Previous knowledge about the NOX2-mediated redox signaling in aggravating tissue inflammation

Accumulating evidence reveals a critical distinction between the local and systemic roles of NOX2 in disease development. Localized oxidative bursts in different tissue microenvironments may drive distinct tissue injury via oxidative modification of cellular molecules, mitochondrial dysfunction and the activation of inflammatory transcription factor (5, 29, 30). Therapeutic strategies have therefore been developed to inhibit NOX2-derived ROS production and limit local tissue damage. Some noted examples of those reports are discussed here.

In ischemic stroke, NOX2 expressed in brain endothelial cells acts as a critical mediator of blood-brain barrier (BBB) disruption. Under conditions of hypoxia and reoxygenation, the cytosolic subunit Rac1 translocates to the plasma membrane to initiate NOX2 assembly and superoxide generation. This oxidative burst triggers the activation of matrix metalloproteinases 9 (MMP-9), which degrade tight junction complexes (31). Inhibition of NOX2 has been shown to attenuate these pathways and preserve BBB integrity (32, 33). In mouse models of Alzheimer’s disease (AD), amyloid-beta (Aβ) deposits trigger persistent microglial NOX2 activation, leading to chronic oxidative stress. Short-term pharmacological blockage of NOX2 effectively inhibits neuroinflammation and prevents behavioral deficits (34). Similarly, evidence from both Parkinson’s disease (35) patients and mouse models indicates that aberrant NOX2 activity in both microglia and neurons mediates oxidative stress-related post-translational modifications of α-synuclein and activates LRRK2, contributing to dopaminergic neurodegeneration; the administration of specific NOX2 inhibitors has been shown to block these downstream toxicities and rescue dopaminergic neurons from cell death (36).

In experimental mouse models of hepatic fibrosis induced by carbon tetrachloride (CCl_4_) administration or bile duct ligation (BDL), NOX2 activation in Kupffer cells and hepatic stellate cells drives oxidative stress that promotes hepatic fibrogenesis; indeed, NOX2 deficiency significantly reduces reactive oxygen species generation, collagen deposition, and liver scarring (37). Studies of environmentally induced lung injury further indicate that NOX2 hyperactivation serves as a pivotal trigger for maladaptive tissue remodeling. In elastase-induced emphysema, NOX2-derived ROS from macrophages lead to the downregulation of sirtuin 1 (SIRT1) levels, which subsequently triggers the upregulation of matrix metalloproteinase-9 (MMP-9) expression and activity, directly resulting in the proteolytic degradation of alveolar elastin (38). Meanwhile, in silica-induced lung injury, NOX2 signaling sustains a deleterious positive feedback loop, in which macrophages promote inflammation through chemoattractant production and neutrophils exacerbate tissue damage through neutrophil extracellular trap (NET) formation; notably, disrupting this phagocytic crosstalk with NOX2 inhibitors effectively mitigates the pulmonary impairment (39).

In summary, these findings and many other studies appear to support the paradigm that NOX2-derived ROS aggravate tissue inflammation and inhibiting NOX2 is therapeutically beneficial for limiting oxidative tissue damage. In contrast, studies on CGD patients and animal models showed that NOX2 is essential for controlling tissue inflammation and maintaining immune homeostasis. The mechanisms underlying these paradoxical immune responses are discussed in the following sections.

## 2 Aberrant immune reactions driving inflammation and autoimmunity in NOX2 deficiency

**Figure 1:**
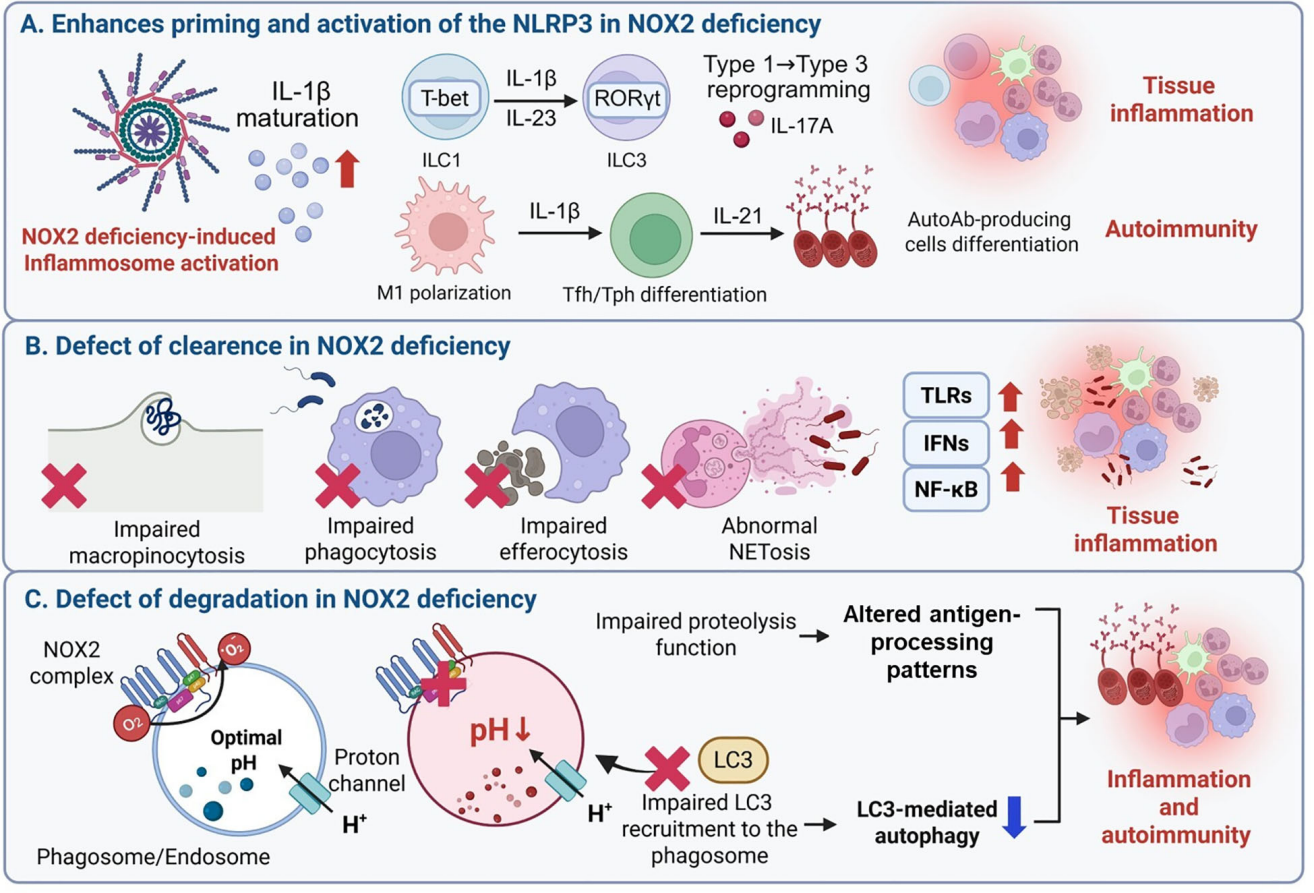
Mechanisms of NOX2 deficiency-induced autoinflammation and autoimmunity. NOX2 deficiency drives autoinflammation and autoimmunity by enhancing both the priming and activation of the NLRP3 inflammasome while impairing the clearance and degradation of pathogens and cellular debris. **(A)** Increased NLRP3 inflammasome activation promotes the release of mature IL-1β into the extracellular space, inducing transdifferentiation of ILC1 into ILC3 and triggering tissue inflammation. In addition, inflammasome activation bridges autoinflammation to autoimmunity through IL-1β-producing macrophages that promote T follicular helper cell differentiation. These T follicular helper cells produce IL-21, which may facilitate the expansion and differentiation of autoreactive B cells. **(B)** Defective clearance in NOX2 deficiency includes impaired macropinocytosis, phagocytosis, efferocytosis, and NETosis, resulting in recurrent infections and prolonged antigen exposure. This persistent stimulation amplifies inflammatory signaling pathways, including TLRs and interferons. Failure to restrain signaling via the apoptotic annexin-driven Dectin-1/NOX2 axis also exacerbates tissue inflammation (71). **(C)** Concurrently, NOX2 deficiency perturbs intracellular degradation pathways, leading to abnormal phagosomal proteolysis, defective LAP, and altered redox-dependent antigen processing that skews immune tolerance toward autoreactivity. Defective LC3 recruitment induces cellular stress, resulting in persistent activation of inflammatory signaling pathways that promote autoinflammation and autoimmune pathology. Moreover, excessive phagosomal acidification alters protease activity and skews epitope selection toward autoreactive determinants, promoting epitope spreading and autoimmune activation. These defects at different subcellular levels link innate redox dysregulation to adaptive immune activation, ultimately driving systemic hyperinflammation and autoimmunity in the context of NOX2 deficiency.

Deficiency of functional NOX2 not only causes CGD but also predisposes affected individuals to systemic hyperinflammation and autoimmunity. Mechanistically, NOX2 deficiency promotes excessive IL-1β production through both inflammasome-dependent and inflammasome-independent pathways, linking impaired ROS production to tissue inflammation and facilitating the transition to autoimmunity (40, 41). Concurrent defects in pathogen and apoptotic cell clearance, including impaired phagocytosis and efferocytosis, result in prolonged exposure to pathogens, cellular debris, and autoantigens (42–45). Loss of NOX2-derived ROS disrupts proteolytic conditions, impairing pathogen clearance, autophagy, and antigen processing, thereby skewing epitope presentation toward autoreactivity and promoting autoimmune inflammation (27, 46–51). Furthermore, while NETosis is diminished under NOX2-deficient conditions, the process exhibits distinct qualitative changes, demonstrating atypical proinflammatory characteristics that intensify immune activation (52–54). Overall, these deviations contribute to sustained local and systemic immune dysregulation as summarized in **Figure 1**. The aberrant immune responses starting at the innate immune cell levels are discussed in 3 categories in the following sections.

### 2.1 Increased IL-1β production connecting phagocyte NOX2 deficiency to inflammation and autoimmunity

CGD patients and NOX2-deficient models showed heightened NLRP3-caspase-1-IL-1β activation, associated with inflammatory bowel disease (IBD), anti-neutrophil cytoplasmic antibody (ANCA)-associated glomerulonephritis, and atherosclerosis (55–57). The enhanced IL-1β production observed in NOX2 deficiency arises through both NLRP3-dependent and NLRP3-independent pathways.

In the NLRP3-dependent pathway, functional NOX2 normally acts as a critical negative regulator of inflammasome activation. The well-known process of IL-1β maturation includes the initial priming by pathogen-associated molecular patterns (PAMPs) or danger-associated molecular patterns (DAMPs) (Signal 1) to induce pro-IL-1β production, followed by the caspase-1-mediated cleavage through activation of the NLRP3 inflammasome (Signal 2). Notably, NOX2-deficient phagocytes, including monocytes and neutrophils from CGD patients, display mitochondrial damage characterized by increased mitochondrial mass, and elevated mitochondrial ROS production, which promote NLRP3 inflammasome assembly and activation of caspase-1. Activated caspase-1 subsequently cleaves pro-IL-1β into mature IL-1β, leading to its extracellular release (56). This regulatory function is mediated by NOX2-derived superoxide, which fine-tune inflammasome activation at multiple levels. Evidence from SOD1-deficient macrophages demonstrated that elevated superoxide inhibits caspase-1 activity through reversible oxidation and glutathionylation of redox-sensitive cysteine residues (C397 and C362), thereby reducing IL-1β maturation and attenuating endotoxic shock (58).

Beyond the inflammasome-dependent pathway, NLRP3-independent mechanisms also contribute substantially to IL-1β generation in NOX2 deficiency. Our previous work by Huang et al. revealed that superoxide inhibits cathepsin B, a lysosomal cysteine protease involved in both NLRP3-dependent and independent activation of pro-IL-1β. Inhibition of caspases and cathepsins efficiently lowered the IL-1β levels in the NOX2-deficient arthritic mice, highlighting the contribution of upregulated protease activity to the IL-1β dominant inflammation (40). Consistent with this concept, IL-1 antagonists reduced arthritis severity selectively in NOX2-deficient mice, indicating that IL-1β drives tissue inflammation (41). CGD granulomas are characterized by massive neutrophil infiltration, which leads to the release of lysosomal serine proteases, including proteinase 3 (PR3) and cysteine proteases, including cathepsin B, which are reported to be active in processing pro-IL-1β to IL-1β (59–61). Furthermore, pathogen-derived proteases can also cleave secreted pro-IL-1β into mature IL-1β (40). Together, these host-derived and pathogen-derived proteolytic pathways create an NLRP3-independent mechanism for higher IL-1β generation in NOX2 deficient conditions.

Being one of the most pro-inflammatory cytokines, IL-1β signaling can further exacerbate inflammation by remodeling innate immune cell networks. A key mechanistic bridge linking phagocyte redox defects to tissue-level inflammation is the ability of IL-1β to remodel innate lymphoid cell (ILC) phenotypes and functions. In a prior work by Chan et al., we showed that NOX2-deficient inflammatory arthritis is marked by elevated IL-1β, which is associated with ILC3 expansion and aggravated joint inflammation. Mechanistically, *ex vivo* IL-1β stimulation of joint-derived T-bet^+^ ILC1s promoted their differentiation into RORγt^+^ ILC3s, supporting the concept that NOX2 functions as a redox regulator of ILC plasticity during inflammation (see Section 3.3 for further details). Activated ILC3s, driven by IL-1β and IL-23 under inflammatory conditions, produce IL-17 and IL-22, which enhance neutrophil recruitment and exacerbate tissue damage (41). Consistent with this mechanism, studies of pulmonary inflammation indicate that cytokine-dependent ILC3 activity contributes to inflammatory lung phenotypes, suggesting that an IL-1β-ILC3 axis may operate across tissues under conditions of chronically elevated IL-1β signaling (62). At the effector level, NOX2 regulates neutrophil inflammatory activity beyond its role in oxidant-mediated microbial killing. In arthritis models, NOX2-deficient neutrophils display enhanced pro-inflammatory responses and reduced regulatory activity, promoting feed-forward inflammation (63). These observations support the existence of an IL-1β-ILC3/type 3 cytokine (IL-17/IL-22)-neutrophil inflammatory circuit that is normally restrained by NOX2-dependent redox regulation (40, 64).

Beyond amplifying innate inflammatory circuits, excessive IL-1β signaling can also promote adaptive autoimmune responses. Under physiological conditions, NOX2-derived ROS, particularly hydrogen peroxide, function as key regulatory signals that restrain IL-1β production in immune cells (65). Loss of this redox control therefore results in exaggerated NLRP3 inflammasome and cathepsin B activation which facilitates IL-1β production. Notably, NLRP3 signaling components are expressed in both innate and adaptive immune compartments which amplifies IL-1β-driven pro-inflammatory immune polarization and promotes autoantibody production (66–68). In the context of NOX2 deficiency, excessive IL-1β may drive autoreactive B cell differentiation and expansion, thereby enhancing autoimmune responses. Mechanistically, NLRP3/IL-1β signaling may promote germinal center responses through both B cell-intrinsic activation and extrinsic signals derived from macrophages and T follicular helper (Tfh) cells (69, 70). These potential pathways collectively implicate a link between innate inflammasome activation and adaptive autoimmunity in the setting of NOX2 deficiency. Further experimental and clinical evidence is needed to solidify this mechanistic link.

### 2.2 Defective clearance as a driver of autoinflammation and autoimmunity in NOX2 deficiency

Defects in phagocytic clearance through endocytic pathways, NOX2 deficiency has been shown to impair macropinocytosis of antigens and lipids, as well as efferocytosis of cellular debris, leading to chronic tissue inflammation and prolonged autoantigen exposure thereby promoting autoimmune responses. Previous studies have demonstrated that NOX2 regulates membrane ruffle formation and macropinocytosis in macrophages through phosphatase inactivation, cofilin dephosphorylation, and activation of the PI3K/Akt pathway, thereby promoting lipid uptake (42).NOX2 also promotes HGF- and PMA-induced macropinocytosis of ovalbumin (OVA) in immature dendritic cells via PKCδ activation, as evidenced by significantly reduced uptake of fluorescently labeled OVA in NOX2-deficient immature dendritic cells (iDCs) compared with wild-type cells (43). In addition, NOX2-derived ROS are required for the oxidation of cell surface molecules such as phosphatidylserine (PtdSer) on apoptotic cells, converting it into an “eat-me” signal. In the absence of this signal oxidation, macrophages exhibit defective efferocytosis, leading to the accumulation of secondary necrotic debris (44). Additionally, dendritic cell Dectin-1 recognizes apoptotic cell annexins A1, A5, and A13, phosphorylating spleen tyrosine kinase (SYK) to activate NOX2. The resulting ROS production suppresses NF-κB-mediated pro-inflammatory cytokine release, thereby maintaining immune tolerance, a process disrupted in NOX2 deficiency (71).

Beyond defects in macrophage- and dendritic cell-mediated endocytic clearance, NOX2 deficiency also disrupts neutrophil extracellular trap formation and skews NETosis toward a proinflammatory phenotype, further sustaining inflammatory stimuli. NETosis normally releases chromatin structures decorated with cytotoxic granule proteins and histones to trap and eliminate pathogens (72, 73). In NOX2-deficient neutrophils, NET formation is markedly reduced, as demonstrated in PMA-stimulated cells from CGD patients, resulting in impaired pathogen clearance and recurrent infections (54, 74, 75). Notably, although reduced in abundance, the residual NETs display a heightened inflammatory phenotype characterized by increased histone hypercitrullination and a prominent type I interferon (IFN) gene signature, which compromises pathogen clearance and exacerbates inflammation in CGD patients (52). Additionally, NOX2 deficiency shifts canonical NETosis toward a mitochondria-derived ROS-dependent pathway, which may enhance ERK1/2 MAPK activation and promote proinflammatory cytokine production (53, 76). Consistently, NOX2 agonist treatment restored NET formation and ameliorated lupus nephritis in lupus mice by limiting inflammatory cytokine release (77).

These defects in clearance lead to the accumulation of pathogens and self-antigens, which activate TLRs, type I IFNs, inflammasome pathways, and downstream inflammatory signaling cascades (78, 79). Persistent activation of these pathways drives tissue inflammation and promotes autoantibody production, ultimately contributing to autoimmune diseases such as systemic lupus erythematosus (SLE) in NOX2-deficient individuals (77, 80, 81).

### 2.3 Impaired intracellular degradation and aberrant autoantigen processing in NOX2 deficiency

NOX2-derived ROS are required for LC3-associated phagocytosis (LAP), a process critical for degradation of phagocytosed material. Under basal conditions NOX2-derived ROS are required to recruit the autophagosome marker LC3 to the phagosome membrane to facilitate the fusion with lysosomes (82). In NOX2-deficient myeloid cells, the failure of LC3-associated phagocytosis prevents the degradation of phagocytosed agonists and damaged mitochondria. The resulting accumulation of cytosolic DNA and mitochondrial ROS (mtROS) acts as a second signal to trigger the assembly of the NLRP3 inflammasome (46). This aberrant pathway activation leads to the cleavage of pro-IL-1β into active interleukin-1β, driving the systemic autoinflammatory granulomas characteristic of CGD (83, 84). Similarly, in *Ncf1^−/−^* marginal zone B cells, defective LC3 recruitment to early endosomes disrupts normal endolysosomal maturation and delays lysosomal fusion following TLR7 engagement. As a result, TLR7-containing endosomes persist in a signaling-competent state, leading to sustained NF-κB nuclear retention and amplified NF-κB-dependent signaling, which exacerbate autoimmunity (47).

Moreover, NOX2 controls antigen processing by regulating phagosomal pH and protease activity. Under homeostatic conditions, NOX2 is recruited to early phagosomes following phagocytosis, where it counterbalances V-ATPase-mediated proton influx and prevents excessive acidification. This redox buffering maintains an optimal phagosomal pH, ensuring controlled proteolysis, preserving a balanced peptide repertoire for cross-presentation, and generating peptides of appropriate length for class I- and class II-MHC loading (27, 85–88). Additionally, NOX2-derived ROS modulate antigen processing by inhibiting redox-sensitive cysteine proteases and disulfide-bond reduction within antigen-processing compartments (89). Loss of NOX2 may enhance cysteine cathepsin activity and alter proteolysis, thereby reshaping the peptide repertoire available for MHC-I- and MHC-II-mediated presentation. Such altered processing may reduce the presentation of dominant epitopes while favoring the exposure of cryptic self-epitopes, potentially promoting epitope spreading, autoreactive T-cell activation, and increased susceptibility to autoimmunity (48–50, 90–92).

In this context, NOX2-derived ROS selectively regulate phagosomal protease activity. NOX2-derived ROS inhibit cysteine proteases such as cathepsins B and L by disrupting the reductive environment of the phagosomal lumen, while having less effect on aspartic proteases such as cathepsins D and E (88, 90). In NOX2 deficiency, heightened cysteine cathepsin activity accelerates phagosomal proteolysis, leading to altered fragment patterns and shifts in the abundance of distinct peptide epitopes derived from antigens such as hen egg white lysozyme (HEL) and myelin oligodendrocyte glycoprotein (MOG) (48). This altered antigen processing has functional consequences in autoimmune models. In glucose-6-phosphate isomerase (GPI)-induced arthritis, NOX2 deficiency enhances presentation of the arthritogenic hGPIc-c peptide, a disulfide-bond-containing autoantigen that requires reduction for immune recognition. This is partly mediated by increased expression of IFN-γ-inducible lysosomal thiol reductase (GILT) which promotes autoantigen processing and worsens disease severity (49). Consistent with this finding, NOX2-deficient p40^phox^-impaired human B cells exhibit reduced cytoplasmic and MHC class II-restricted exogenous antigen presentation but increased presentation of membrane-associated autoantigens, indicating that NOX2 regulates epitope selection by MHC class II molecules (90). Epitope spreading, defined as the progressive diversification of T cell or antibody responses to additional epitopes within the same or different antigens, is driven by inflammation, tissue injury, and B cell-mediated antigen presentation (93, 94). Importantly, NOX2-derived ROS have been shown to limit intramolecular B cell epitope spreading to Collagen type II (CII) epitopes and to restrain T cell activation, thereby protecting susceptible B10Q.ACB mice harboring germline-encoded CII-specific IgH from exacerbated collagen-induced arthritis (50). Collectively, these results indicate that NOX2 serves as an essential redox regulator for antigen processing, protease activity, and epitope selection. A deficiency in NOX2 alters antigen presentation towards autoreactivity, facilitating epitope spreading and exacerbating autoimmune pathology.

## 3 CGD-associated tissue inflammation and comorbidities

Although patients with CGD caused by NOX2 deficiency are classically characterized by recurrent severe infections (95, 96), increasing evidence demonstrates that both CGD patients and carriers are highly susceptible to autoinflammatory and autoimmune diseases (15, 17, 97–99). Autoinflammatory manifestations include pneumonitis, sterile granulomatous inflammation, IBD, dermatitis, and cardiovascular disease (particularly atherosclerosis) (17, 55, 95, 100, 101). Autoimmune manifestations, including juvenile idiopathic arthritis (JIA)-like disease and lupus-like syndromes such as discoid lupus erythematosus (DLE) and SLE, have also been reported (97, 102–107). As these manifestations have been detailed in many clinical reports, we focus our discussion on mechanism-relevant and organ-specific inflammation in the lungs, cardiovascular system, joints, and lupus-like diseases.

### 3.1 Pulmonary diseases

Emerging evidence suggests that immunoregulatory defects associated with NOX2 deficiency are central to the development of progressive lung injury and pulmonary vascular complications in CGD. The lung is affected in 40-85% of CGD patients, both children and adults (108). Severe pulmonary fibrosis, manifesting as “honeycomb lung”, was reported as an unusual presentation caused by CGD in the 1970s (109). The mechanism underlying this granulomatous inflammation remains incompletely understood. In the context of NOX2 deficiency, patients are susceptible to recurrent pulmonary infections, which can contribute to initiate the chronic lung inflammation. Over time, this persistent inflammatory state may lead to progressive fibrosis of the lung tissue (110). Importantly, evidence from a murine model of NOX2 deficiency have demonstrated that dysregulated inflammatory responses can exacerbate chronic pulmonary inflammation, resulting in sustained neutrophil recruitment, enhanced production of pro-inflammatory cytokines, and persistent granulomatous lesion formation (111).

While infections such as aspergillosis and Staphylococcus are well-known causes of lung damage in CGD, non-infectious manifestations are common and often underdiagnosed (112). During infection-induced inflammation, IFN-γ and TNF-α stimulate the expression of indoleamine 2,3-dioxygenase (IDO), which catalyzes the degradation of tryptophan through the kynurenine pathway. Because many pathogens rely on host-derived tryptophan for protein synthesis, metabolic activity, and replication, IDO-mediated depletion of intracellular tryptophan creates a nutrient-restricted environment that suppresses microbial proliferation and contributes to host antimicrobial defense (113–118). Importantly, optimal activity of the kynurenine pathway has been suggested to be influenced by NOX2-derived ROS. As kynurenine interacts with aryl hydrocarbon receptor (AhR) in immune cells including macrophages, dendritic cells (DCs), and regulatory T cells (Tregs) to drive IL-10 production, dysfunction of the kynurenine pathway has been observed in murine models of pulmonary aspergillosis, leading to dysregulated IL-17 responses, defective regulatory T-cell activity, and severe inflammatory lung injury, suggesting that disruption of this NOX2-kynurenine axis contributes to CGD-associated hyperinflammation and granulomatous pathology (119–121).

In systemic inflammatory response syndrome (SIRS), functional NOX2 is required to limit neutrophil infiltration and activation of neutrophils and macrophages in the lungs. The absence of NOX2 results in failure to resolve inflammation, leading to progressive lung injury and the development of multiple organ dysfunction syndrome (MODS) in mouse models (122). Neutrophils produce excessive leukotriene B4 (LTB4), a major chemoattractant, to recruit more neutrophils in the lungs of NOX2-deficient mice. The accumulated neutrophils produce a large amount of IL-1β in CGD lungs and result in the downstream increment of granulocyte colony-stimulating factor (G-CSF). The hyperactive IL-1β/G-CSF axis then drives pathological emergency of granulopoiesis and the subsequent influx of specific immature CD101^−^ neutrophil subsets from the bone marrow into the lungs (123). These immature neutrophils may help to sustain the inflammatory milieu leading to pulmonary fibrosis.

Clinically, the cumulative impact of this unrestrained inflammation and fibrosis manifests as distinct vascular pathologies. It was even reported that pulmonary hypertension in CGD patients may mimic pulmonary veno-occlusive disease (PVOD), characterized by the diffuse infiltration of pigmented macrophages into the perivascular interstitium, subpleural tissue and interlobular septa (101). In a case report of autosomal recessive CGD, pulmonary hypertension served as a critical presenting feature of this immunodeficiency, aggravated by fungal infections of *Aspergillosis* (124). Furthermore, large retrospective cohort analyses have corroborated that pulmonary hypertension is predominantly a sequela of the CGD, correlating strongly with long-term pulmonary damage, including extensive fibrosis, mediastinal lymphadenopathy, and pleural thickening (125). Taken together, these results show that chronic inflammatory stress from repeated infections, as well as disruptions in immune regulation caused by ROS, both play a role in the development of lung problems associated with CGD.

### 3.2 Cardiovascular diseases

Although oxidative stress is a well-established driver of endothelial dysfunction, clinical evidence from CGD patients showed that NOX2 activity appears to play a protective role in cardiovascular homeostasis, rather than a deleterious one. An NIH study assessed the atherosclerotic incidence in CGD patients using magnetic resonance imaging and computed tomography. They also found that individuals with CGD have higher levels of cardiovascular risk factors and inflammatory markers, including hypertension, high-sensitivity C-reactive protein (hs-CRP), increased oxidized low-density lipoprotein (LDL), and reduced high-density lipoprotein (HDL), compared with the general population (55). In addition, CGD patients exhibited a significantly increased risk of ischemic coronary and cerebrovascular events compared to the general population (126). To investigate the biological mechanisms driving this increased cardiovascular risk, experimental models were employed using total genetic ablation and pharmacological inhibition approaches. In a murine model of insulin resistance, pharmacological inhibition of NOX2 reduced atherosclerosis, whereas total genetic deletion of NOX2 paradoxically accelerated atherosclerotic plaque formation. Specifically, genetic NOX2 ablation led to the disruption of aortic elastic laminae with increased thoracoabdominal atherosclerosis. This indicates that basal NOX2 activity is required to maintain aortic wall integrity and reduce the risk of atherogenesis; complete absence of NOX2 predisposes vessels to structural instability and plaque formation (127). In addition to maintaining vascular wall integrity, the protective role of NOX2 activity extends to the myocardium, specifically in maintaining cardiomyocyte viability. NOX2-derived ROS are crucial for cell adhesion receptor CD29 (β1-integrin)-mediated pro-survival signaling in cardiomyocytes. Moreover, the activation of CD29 triggers NOX2 to generate superoxide and hydrogen peroxide, which acts as second messengers to phosphorylate and activate downstream survival pathways, including MEK/ERK and PI3K/Akt (128). The absence of NOX2 disrupts this signaling axis, rendering cardiomyocytes more susceptible to apoptosis.

In addition to its role in maintaining vascular wall integrity and cardiomyocyte survival, NOX2 plays a crucial part in modulating macrophage function, specifically in efferocytosis, an essential process of clearing apoptotic cells. In the context of atherosclerosis, efferocytosis acts as a primary protective mechanism; however, when this process is impaired, the uncleared apoptotic cells undergo secondary necrosis, driving the formation of a necrotic core and exacerbating the inflammatory microenvironment of advanced atherosclerotic plaques (129, 130). Mechanistically, NOX2 serves as a critical orchestrator of this protective clearance. Efficient apoptotic cell clearance often relies on non-canonical autophagy pathways, e.g. LC3-associated phagocytosis, which intimately depend on localized ROS generated by NOX2 to function correctly and prevent inflammatory responses (see Section 2.3) (131, 132). Consequently, in the absence of functional NOX2, macrophages and monocytes exhibit a markedly impaired ability to clear apoptotic debris (133, 134). This genetic ablation of NOX2 not only impairs the engulfment of dying cells by professional phagocytes but also alters macrophage programming, shifting them away from a pro-resolving state (135). Therefore, NOX2 deficiency accelerates atherosclerotic plaque progression and instability in CGD patients through defective myeloid phagocyte efferocytosis.

### 3.3 Arthritis

Patients with CGD have been reported to develop both septic arthritis and non-infectious, autoimmune-like arthritis (99, 105, 106, 136). In a large European CGD cohort study involving 429 patients, approximately 8% experienced at least one episode of proven or suspected infectious arthritis (136). Meanwhile, autoimmune-like arthritis has been particularly associated with mutations in *Ncf1*, with several case reports describing this manifestation and an estimated prevalence of approximately 10% among CGD patients carrying *NCF1* mutations (99, 106).

Animal studies also demonstrated that NOX2 deficiency in arthritic mouse models result in a more severe disease phenotype than wild-type mice, characterized by pronounced synovial inflammation with increased infiltration of pro-inflammatory neutrophils, enhanced joint swelling, progressive cartilage and bone destruction, and elevated autoantibody production (40, 41, 63, 137–140). Consistent with this finding, several arthritis models demonstrated exacerbated joint inflammation in *Ncf1*-deficient mice. In innate immune-driven zymosan-induced arthritis (ZIA), *Ncf1* deficiency leads to aggravated synovial inflammation, irreversible cartilage damage and increased expression of IFN-γ, TNF-α, and IL-1α (137). Similarly, in T cell-mediated collagen-induced arthritis (CIA), *Ncf1*-deficient B10.Q mice developed more severe and chronic relapsing disease, even under specific pathogen-free conditions, accompanied by enhanced IFN-γ and IL-17 production by CII-specific T cells and elevated anti-CII antibody responses (141). Meanwhile, in T and B cell-independent collagen antibody-induced arthritis model, mannan-enhanced type II collagen antibody-induced arthritis (mCAIA) BQ.*Ncf1* ^m1j/m1j^ mice developed chronic relapsing arthritis with joint erosions, marked infiltration of CD11b^+^Ly6G^+^ neutrophils, and sustained elevation of pro-inflammatory cytokines including G-CSF and TNF-α (140). Notably, NOX2-deficient mice were shown to develop spontaneous arthritis in an age-dependent manner. This arthritis is associated with increased TNF-α and IL-1β expression, elevated serum anti-type II collagen IgG levels, expansion of CD11b^+^Gr-1^+^ myeloid cells, with their CD4^+^ T cells showing enhanced IFN-γ and IL-17 production, reduced regulatory T cell (Treg) numbers, and skewing toward Th17 differentiation with aging (139).

Our previous studies demonstrated increased severity of the K/BxN serum-transfer arthritis (STA) in *Ncf1* ^m1j^ and *Cybb*^−/−^ NOX2-deficient mice, characterized by neutrophil-dominant tissue inflammation and elevated expression of IL-1β, IL-17, and IL-6. In the K/BxN STA model, transferring arthritic serum from K/BxN mice to naïve mice mimics the effector phase of RA, driven by autoantibodies against the self-antigen GPI (142). Mechanistic analysis of IL-1β signaling showed that although active cathepsin B and caspase-1 were elevated in arthritic joints, cysteine cathepsins were the dominant drivers of disease exacerbation in NOX2-deficient mice. Importantly, we showed that hydrogen peroxide directly inhibits cathepsin B protease activity during the processing of human pro-IL-1β into its active form (40). In this context, elevated IL-1β levels in NOX2-deficient arthritic joints promote the trans-differentiation of ILCs toward natural cytotoxicity receptor-positive NCR^+^ ILC3s which promote type 17 immune responses. Treatment with the IL-1 receptor antagonist anakinra reduces the proportion of NCR^+^ and IL-17A-producing ILC3s in arthritic tissues and alongside ameliorates joint inflammation (41). These findings support that NOX2-derived ROS restrain IL-1β-mediated inflammation and identify tissue ROS levels as a critical determinant of arthritis severity. Our previous work by Liao et al. also identified NOX2-deficient neutrophils as critical contributors to the exacerbation of inflammation in K/BxN serum transfer arthritis in *Ncf1* ^m1j^ mice. Neutrophils lacking NOX2 from inflamed joints exhibit hyperinflammatory phenotype characterized by elevated expression of pro-inflammatory genes, including *Il1b*, *Cxcl2*, *Cxcl3*, *Cxcl10*, and *Mmp3*, and enrichment of type 1and 2 interferons, IL-6-JAK-STAT3, and NF-κB-TNF-α signaling pathways. Moreover, NOX2-deficient neutrophils express lower levels of PD-L1 and display a reduced capacity to suppress T cell proliferation while PD-L1-Fc treatment restores immune regulation and ameliorates disease severity which is a potential treatment target of abnormal redox regulation immune-mediated arthritis (63).

### 3.4 Lupus-like disease

CGD patients with autoimmune manifestations frequently develop lupus-like diseases, with a high prevalence of DLE and SLE (102, 103, 107, 143–146). Indeed, polymorphisms in *NCF1* and *NCF2* have been associated with an increased risk of autoimmune diseases, particularly SLE, with high odds ratios indicating a strong effect on disease susceptibility. These associations have been identified through genome-wide association studies (GWAS), meta-analyses, fine-mapping, and replication studies (147–152). Among lupus patient who have the SNP *NCF1*-339 0C genotype, impairs canonical NOX2-dependent NETosis and shifts neutrophils toward mitochondria-derived ROS-dependent NET formation which is strongly associated with heightened interferon responses and antiphospholipid antibody production, contributing to lupus progression and antiphospholipid syndrome in carriers of the *NCF1*-339 0C genotype (151). Consistently, reduced granulocyte NOX2-derived ROS production is associated with lupus development, as impaired neutrophil ROS generation negatively correlated with the erythrocyte sedimentation rate (ESR), anti-dsDNA levels, and overall disease activity, supporting a protective role for NOX2-derived ROS (153, 154). Consistent with the immunoregulatory role of NOX2-derived ROS, NOX2 deficiency exacerbates SLE pathogenesis across diverse murine models. Both spontaneous and inducible lupus models deficient in NOX2 consistently demonstrate accelerated disease development and amplified clinical severity (47, 77, 155, 156).

Defective clearance of apoptotic material in NOX2 deficiency sustains immune activation and prolongs exposure to autoantigens and inflammatory cytokines, thereby exacerbating lupus-associated inflammation and autoimmunity. Monocytes and neutrophils from CGD patients accumulate secondary necrotic cells due to delayed phagosomal degradation, leading to enhanced release of proinflammatory cytokines, including TNF-α, IL-1β, and CXCL1 (81). Additionally, impairment of the annexin/Dectin-1/NOX2 axis disrupts NF-κB inhibition, further triggering the TNF-α, IL-6, and IL-12 production (71). Persistent autoantigen exposure further promotes overactivation of TLR7/9 and type I interferon signaling, fostering autoreactive B-cell differentiation (157–159).

Meanwhile, amplified type I interferon responses further drive disease progression. Increased interferon-stimulated gene (ISG) expression correlates positively with autoantibody titers and autoreactive B-cell expansion (159–162). Both CGD patients and *Ncf1*-deficient mice display an elevated interferon signature (52, 163). In *Ncf1*-mutant mice with NOX2 deficiency, diminished ROS production in plasmacytoid dendritic cells (pDCs) facilitate their CCR2-dependent recruitment to multiple organs and potentiates activation of the STING-IFN-α-JAK1-STAT1 pathway. At the same time, excessive endosomal acidification enhances TLR activation and downstream signaling, amplifying pathogenic type I interferon responses. Importantly, restoring ROS levels in pDCs attenuates lupus nephritis and ameliorates disease severity (152, 156). Alongside this, environmental triggers such as viral infections can establish a feed-forward IFN-TLR7 circuit, enhancing germinal center formation and pathogenic autoantibody production (164). These findings support impaired NOX2-derived ROS production as a critical contributor to lupus pathogenesis.

## 4 Type 1 immune responses in NOX2 deficiency

Patients and animal models with NOX2 deficiency exhibit exaggerated type 1 immune responses, characterized by increased expression of Th1-associated inflammatory genes and elevated production of Th1-related pro-inflammatory cytokines within lymph nodes and abscess lesions (152, 165, 166). Skewing toward Th1-polarized CD4^+^ T cells and Th1- polarizing cytokine-producing M1 macrophages enhances IFN-γ production, thereby establishing a pathogenic inflammatory microenvironment (152, 165). Although macrophages are highly plastic and adopt diverse phenotypes depending on the local microenvironment, this review uses the classic M1/M2 framework for simplicity, defining M1 macrophages as generally proinflammatory and M2 macrophages as anti-inflammatory (167). This IFN-γ-enriched microenvironment further promotes activation of type I interferon signaling and the IL-1β/NLRP3 inflammasome pathway, thereby driving persistent inflammation and promoting autoimmunity through autoreactive B-cell differentiation and expansion (47, 56, 152, 163). Importantly, the presence of a pre-existing IFN-γ-enriched environment in NOX2 deficiency raises concerns regarding the potential risks of IFN-γ therapy in patients with CGD and related disorders (168).

### 4.1 Enhanced Th1 immune response in CGD patients and animal models

Type 1 immune responses are essential for defense against intracellular pathogens, including viruses, mycobacteria, and parasites, and are characterized by pro-inflammatory activation dominated by IFN-γ-producing Th1 cells that activate macrophages and support effective CD8^+^ cytotoxic T lymphocyte (CTLs) responses. The key cytokines IFN-γ and IL-12 drive Th1 differentiation by activating STAT1 and STAT4, respectively, inducing the transcription factor T-bet, and promoting the production of IFN-γ, IL-2, and TNF-α (169). However, excessive activation of type 1 immune responses partially contributes to the development of autoinflammatory and autoimmune diseases, including atherosclerosis, IBD, type 1 diabetes (TD1), rheumatoid arthritis (RA), lupus, and multiple sclerosis (MS) (169–173).

In NOX2 deficiency, loss of the respiratory burst impairs antimicrobial defense but paradoxically enhances Th1 responses, driving pathological Th1 differentiation and excessive IFN-γ production, thereby increasing susceptibility to autoinflammation and autoimmunity. Consistent with this, previous studies have found that CD4^+^ T helper cells from NOX2-deficient *Cybb*^−/−^ mice exhibit Th1 skewing, characterized by increased expression of the Th1-specific transcription factor T-bet and enhanced IFN-γ production following T cell receptor activation (174, 175).

Beyond T cell-intrinsic mechanisms, enhanced Th1-polarizing cytokines in NOX2-deficient settings drive Th1 skewing. Elevated levels of the Th1 cytokines IL-18 and IFN-γ in inflammatory tissues, including lymph nodes and submuscular thoracic abscesses from CGD patients, have been reported. Macrophages were identified as a major source of IL-18 and persist for up to six weeks in inflamed lymph nodes from CGD patients (165). The IFN-γ inducing factor IL-18 has been identified to facilitate the production of IFN-γ from NK cells and Th1 lymphocytes (176–178). The persistent IL-18 production by M1 macrophages in CGD-associated inflammatory tissues sustains IFN-γ secretion from other immune cells, may thereby amplifying pathological Th1 differentiation and contributing to chronic inflammation and autoimmunity (179). Previous studies have demonstrated that NOX2-deficient dendritic cells produce elevated levels of IL-12, thereby promoting IFN-γ production. Specifically, NOX2-deficient *Cybb*^−/−^ bone marrow-derived dendritic cells (BMDCs) enhance T cell activation when co-cultured with wild-type T cells and exhibit increased IL-12 production in response to Dectin-1 stimulation (166). Similarly, p47^phox−/−^ dendritic cells secrete excessive IL-12p70 following IFN-γ/LPS stimulation and preferentially drive CD4^+^ T cells toward a Th1 phenotype via enhanced p38 MAPK activation (180). Specifically, the absence of NOX2-derived ROS intrinsically skewed naive CD4+ T cells toward Th1 phenotype, while simultaneously paralyzing the development and suppressive capacity of Tregs (174, 181, 182). NOX2-deficient CD4+ T cells exhibited an inherent bias toward Th1 lineages upon primary activation, characterized by augmented IFN-γ production, diminished IL-4 secretion, and an elevated T-bet to GATA-3 transcription factor ratio. This intrinsic skewing was driven by a selective reduction in TCR-induced STAT5 phosphorylation in the absence of ROS. Furthermore, this T cell bias was extrinsically amplified by NOX2-deficient antigen-presenting cells; dendritic cells lacking functional NOX2 produced elevated levels of pro-inflammatory cytokines such as IL-6 and TNF-α, which subsequently promoted exaggerated Th1 and Th17 responses in interacting T cells (174).

A Th1-enriched environment can also foster type I interferon responses through activation of the IFN-JAK-STAT1 signaling pathway (183). Consistent with this, elevated type I interferon signatures, particularly increased STAT1 expression, have been observed in leukocytes from patients with CGD and in NOX2-deficient mice (52, 163). Moreover, the upregulated activation of NLRP3 inflammasome promotes IL-1β secretion also contributes to the Th1 immune response in NOX2 deficiency (56, 65, 184). A prior study demonstrated that autocrine IL-1β signaling driven by NLRP3 inflammasome activation is essential for optimal IFN-γ production by T cells (185). In addition, both IL-1β and IL-18 promote Th1 immune responses by enhancing IFN-γ production and supporting sustained T cell activation which causes more severe inflammation. Meanwhile, B cells themselves are capable of activating the NLRP3-caspase-1 inflammasome and producing IL-1β. Taken together, these processes establish a feed-forward circuit that amplifies germinal center responses and promotes autoantibody production (69, 70, 186–188).

Collectively, these findings identify NOX2-derived ROS as critical secondary messengers that fine-tune cytokine secretion and inflammatory immune crosstalk which correlated with type 1 immunity.

### 4.2 NOX2-dependent regulation of macrophage differentiation in type 1 immunity

Accumulating evidence demonstrates that NOX2-derived ROS function as critical signaling molecules that not only orchestrate M2 macrophage polarization but also actively suppress pro-inflammatory pathways to maintain tissue homeostasis. This essential regulation of macrophage plasticity and inflammation resolution operates through distinct mechanistic pathways. At the level of intracellular signaling, NOX2-derived ROS act as essential secondary messengers that activate the MAP kinases ERK and JNK, which drive monocyte-to-macrophage differentiation and M2 macrophage polarization (189). Research has also shown that NOX2-derived ROS facilitate STAT6 activation through a redox-sensitive cysteine residue at position 528 (Cys528), leading to the upregulation of M2 gene *Fizz1* (190). In addition to actively promoting these reparative traits, NOX2 signaling serves as a necessary brake on inflammatory responses. The binding of pathogen-derived β-glucans to NOX2-activating receptor Dectin-1 on macrophages triggers the secretion of pro-inflammatory markers and cytokines through the activation of the transcription factor NF-κB. However, this response is actively suppressed by NOX2-derived ROS. On the other hand, the recognition of endogenous ligands, such as annexins, activates NOX2 to generate ROS. These NOX2-derived ROS are shown to inhibit NF-κB signaling, thereby limiting cytokine production and preventing hyperinflammation (8). Moreover, NOX2-deficient mice developed activated CD11b^high^ alveolar macrophages whose proinflammatory epigenetic and transcriptional profiles correlated with an upregulated number of alveolar neutrophils, thereby dysregulating alveolar homeostasis (191).

Beyond intrinsic intracellular pathways, the absence of ROS profoundly alters the local immune microenvironment through self-amplifying paracrine signaling. In the absence of ROS-dependent regulatory signal, the balance of macrophage polarization is profoundly skewed toward a pro-inflammatory state. Patients with CGD exhibit a prolonged accumulation of M1 pro-inflammatory macrophages within the chronically inflamed tissues (165). Immunohistochemical analysis identified these M1 macrophages as the primary source of IL-18 in the lesions. A study revealed that these cells are maintained in a state of M1 hyper-activation by a pro-inflammatory positive feedback loop where M1 macrophage-derived IL-18 stimulates infiltrating lymphocytes to secrete high levels of IFN-γ (192), which in turn feeds back onto the macrophages to sustain their M1 polarization and prevent resolution. However, this polarization is not an irreversible intrinsic defect. *In vitro* experiments showed that CGD macrophages retain the plasticity to be re-primed into the anti-inflammatory M2 phenotype when removed from the inflammatory milieu. This suggests that the sterile hyperinflammation in CGD is driven by the environmental lock-in of M1 macrophages within this IL-18/IFN-γ feedback loop, rather than a permanent loss of regulatory potential.

**Figure 2:**
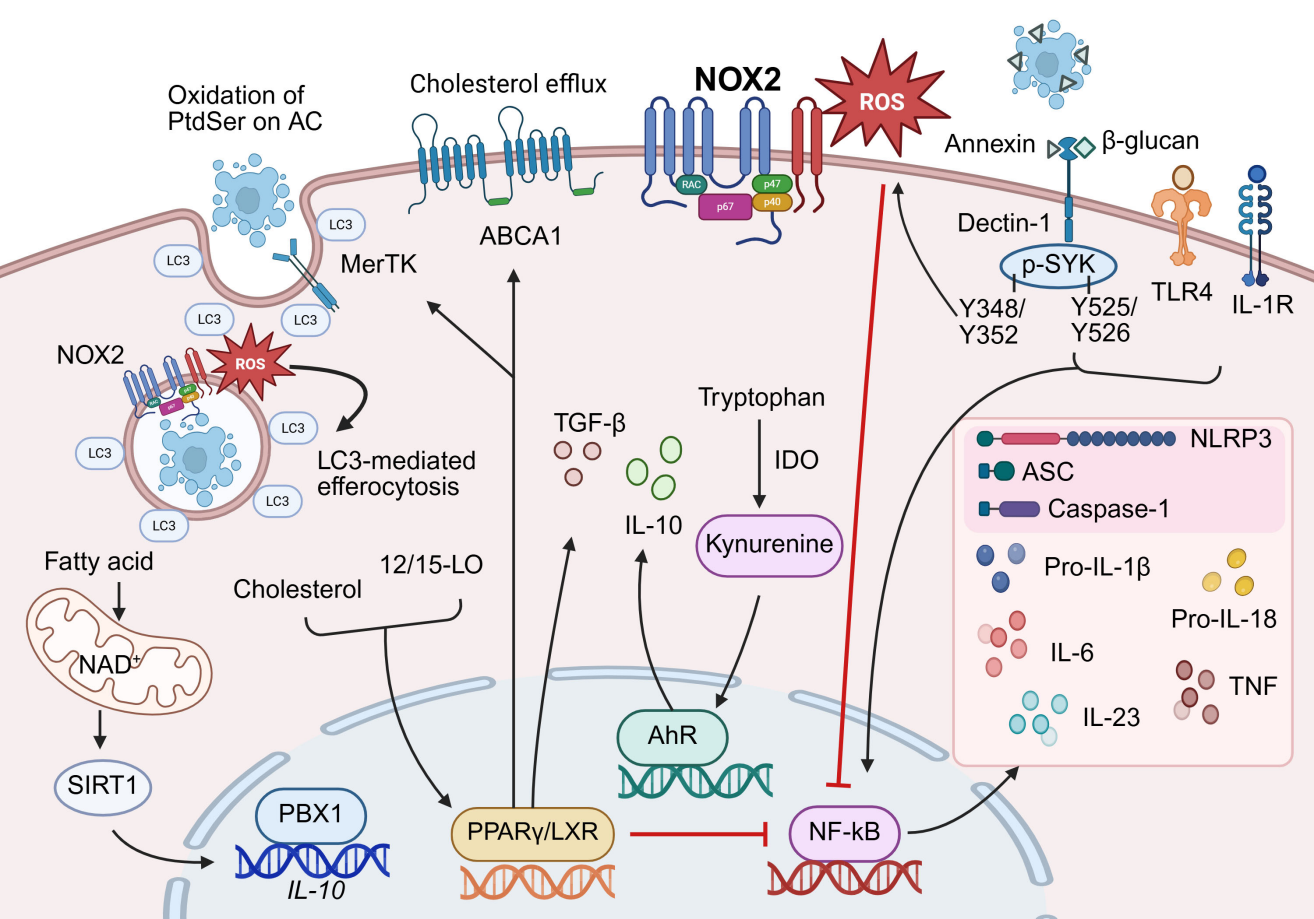
NOX2-mediated NF-κB inhibition drives inflammation resolution in macrophages. NOX2-derived ROS are required for the peroxidation of cell membrane phospholipids, such as PtdSer on apoptotic cells, which enables macrophages to recognize and ingest. The NOX2-derived ROS then triggers LC3 recruitment to the efferosome and followed by digestion of apoptotic cell (AC)-associated cargos including proteins, lipids and nucleic acids. AC-derived fatty acids undergo mitochondrial oxidation to generate NAD^+^, which drives SIRT1 deacetylase to activate PBX1 transcription factor and subsequent IL-10 secretion. The production of NOX2-derived ROS activates IDO, which subsequently catalyzes the conversion of tryptophan to kynurenine. Kynurenine then interacts with AhR to drive IL-10 production. AC-derived cholesterol and 12/15-LO activate nuclear receptors LXR and PPARγ, which increases ABCA1 expression to excrete cholesterol, induces MerTK expression for continuous efferocytosis and promotes resolving cytokine secretion. Activation of LPS/TLR and IL-1β/IL-1R signaling promotes NF-κB activation and induces the expression of pro-inflammatory mediators, including components of the NLRP3 inflammasome and various inflammatory cytokines. Similarly, pathogen-derived β-glucans engage Dectin-1 and induce phosphorylation of distinct tyrosine residues within spleen tyrosine kinase (SYK), including Y525/Y526 and Y348/Y352. While phosphorylation of Y525/Y526 drives NF-κB-dependent inflammatory responses, phosphorylation of Y348/Y352 triggers NOX2-derived ROS production, which serves as a negative feedback mechanism to restrain excessive NF-κB activation. In contrast, endogenous annexins exposed on apoptotic cells bind to a distinct site on Dectin-1 and selectively induce phosphorylation of SYK Y348/Y352, thereby activating the NOX2-ROS-mediated inhibitory pathway that suppresses NF-κB signaling (71). Together with anti-inflammatory signaling mediated by LXR and PPARγ, this pathway promotes tolerogenic phenotypes in antigen-presenting cells (APCs), including macrophages and dendritic cells, thereby maintaining peripheral immune tolerance and preventing excessive inflammation.

Furthermore, this redox-dependent regulation is particularly critical during the physiological clearance of dying cells, a process known as efferocytosis, which orchestrates both intercellular immune circuits and metabolic reprogramming. The efferocytosis of apoptotic neutrophils typically triggers macrophages to upregulate surface CD1d expression and produce IL-4. This signaling activates invariant natural killer T (iNKT) cells, stimulating them to secrete abundant IL-4 and IL-13 that synergize to enforce a phenotypic switch of macrophages from an inflammatory M1 to a reparative M2 state. However, in the absence of functional NOX2, this efferocytosis-induced CD1d upregulation is defective. The consequent failure to activate the macrophage-iNKT cell regulatory circuit arrests macrophages in the hyper-inflammatory M1 phenotype, thereby perpetuating sterile inflammation and exacerbating tissue injury (193). In parallel with this defective intercellular circuit, the transcription factor PPARγ orchestrates the resolution of sterile inflammation in CGD. Under normal conditions, the efferocytosis of apoptotic neutrophils triggers PPARγ activation, which subsequently drives the macrophage phenotype switch from pro-inflammatory M1 to anti-inflammatory M2. However, in NOX2-deficient CGD hosts, this efferocytosis-induced PPARγ signaling is impaired, resulting in defective production of resolving cytokines including TGF-β and IL-10 and prolonged accumulation of M1 macrophages (134). The apoptotic cell-derived tryptophan is converted into kynurenine by ROS-dependent IDO, which activates ligand-activated transcription factor AhR to drive IL-10 production (194). 12/15-lipoxygenase (LO) generates oxidized lipid mediators such as 13-hydroxyoctadecadienoic acid (13-HODE) and 15-hydroxyeicosatetraenoic acid (15-HETE) that serve as endogenous ligands for PPARγ, which then cooperates with IL-4 to enforce the M2 transcriptional program (194). Consequently, the lack of ROS in CGD leads to a combined defect in the synthesis of these specific lipid agonists and IL-4 production, thereby preventing the reprogramming for inflammation resolution (**Figure 2**).

### 4.3 Th1 environment on autoreactive B cell differentiation

NOX2 promotes autoreactive B cells differentiation through both B cell-intrinsic and B cell-extrinsic pathways. For B cell-intrinsic mechanisms, NOX2-deficient B cells have been shown to exhibit enhanced TLR7/9-induced NF-κB and MAPK activation, promoting inflammatory cytokine production, spontaneous germinal center formation, and increased autoantibody production (47, 195, 196). The underlying mechanism involves enhanced downstream signaling of endosomal TLRs in response to nucleic acid-containing antigens, resulting from impaired LC3 recruitment to early endosomes. This defect delays endosome-lysosome fusion, thereby breaking B-cell tolerance in *NCF1*-deficient B cells and promoting autoantibody-producing B-cell formation, extrafollicular age-associated B cells (ABCs) differentiation and the development of autoimmunity (47). In addition, the Th1-enriched microenvironment synergizes with B cell-intrinsic TLR7/9 hyperactivation to drive autoreactive B-cell differentiation in NOX2 deficiency.

**Figure 3:**
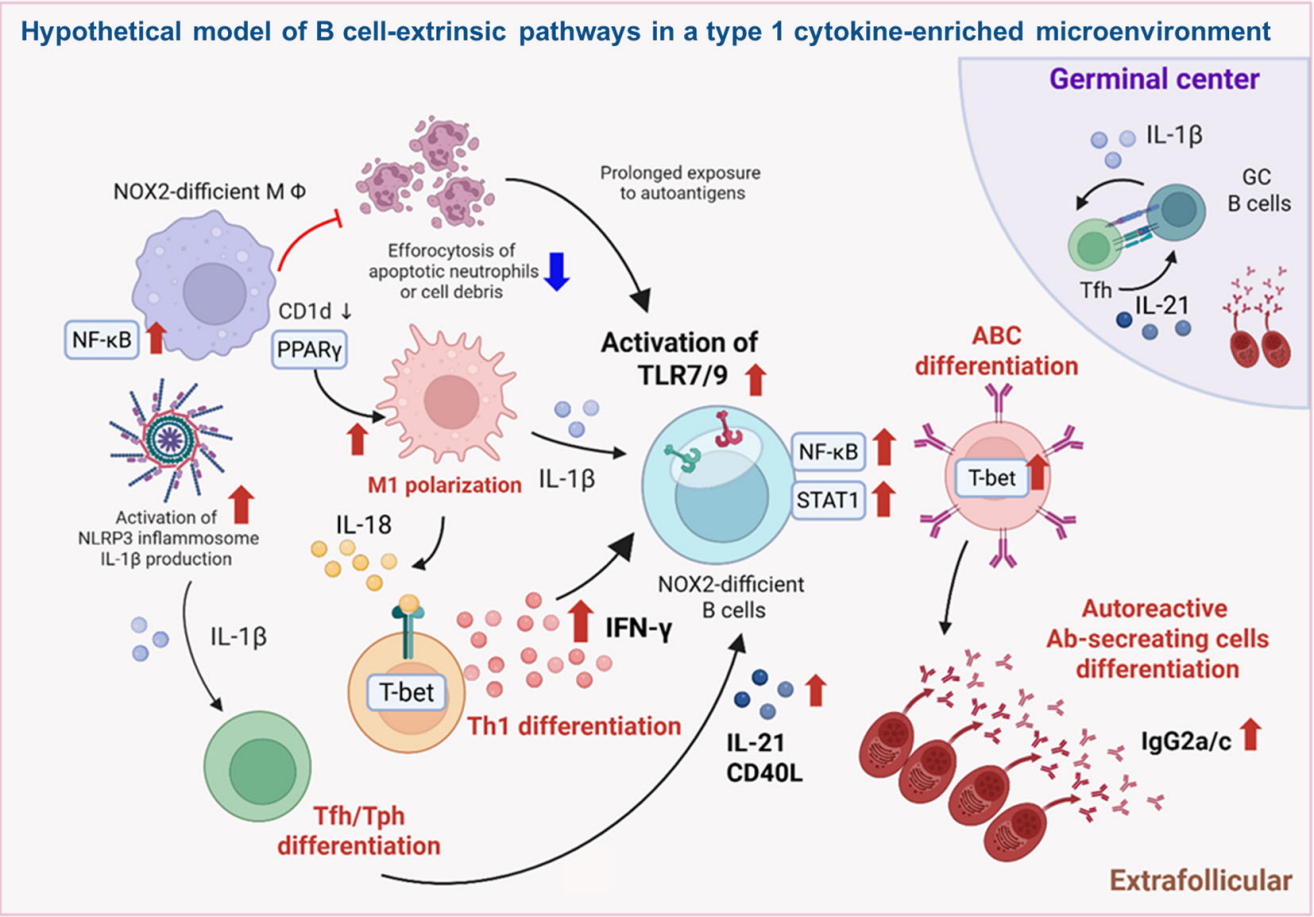
Type 1 immune response-driven autoreactive B cell development in NOX2 deficiency. Defective efferocytosis of apoptotic neutrophils and cellular debris in NOX2-deficient macrophages leads to reduced PPARγ activation and diminished efferocytosis-induced CD1d expression, thereby promoting pro-inflammatory M1 polarization. Impaired clearance further results in prolonged accumulation of autoantigens, which enhances TLR7/9 activation in B cells. M1 macrophages produce elevated levels of IL-18, driving type 1 immune responses and promoting Th1 differentiation and IFN-γ production. Simultaneously, enhanced IL-1β-NF-κB-dependent priming of the NLRP3 inflammasome in NOX2-deficient macrophages promotes maturation and secretion of IL-1β, thereby stimulating B-cell activation and differentiation of T follicular helper (Tfh) and T peripheral helper (Tph) cells (69, 186, 187). The combined effects of hyperactivated TLR7/9 signaling, increased Th1-derived IFN-γ, and Tfh/Tph-derived IL-21 favor the expansion of ABCs and their differentiation into antibody-secreting cells, leading to rapid autoantibody production and preferential IgG2a/c isotype switching through extrafollicular pathways. In addition, within germinal centers, IL-1β-producing B cells further support Tfh differentiation and function, thereby enhancing plasma cell generation (70). Collectively, the Th1- and IL-1β-enriched inflammatory microenvironment in NOX2 deficiency promotes autoreactive B-cell differentiation and drives the development of autoimmunity.

For B cell-extrinsic pathways, a Th1-enriched environment may promote autoreactive B cell formation in NOX2 deficiency. ABCs, also referred to as atypical B cells, have been identified in autoimmune diseases including SLE, RA, Sjogren’s syndrome, and MS (197, 198). ABCs are characterized by enhanced antigen presentation and inflammatory cytokine production, and preferentially generate low-affinity autoantibodies through the rapid formation of extrafollicular plasmablasts and short-lived plasma cells (197, 199, 200). TLR7, IFN-γ, and IL-21 signaling are critical for ABC development (201). Upon recognition of nucleic acid-containing autoantigens, B-cell receptor (BCR) engagement facilitates the internalization and delivery of autoantigens to TLR7-containing endosomes, activating MyD88-dependent signaling and downstream transcription factors including NF-κB, AP-1 and interferon regulatory factors (IRFs) (202). This signaling cascade induces the production of inflammatory cytokines and type I and II interferons, thereby promoting ABC differentiation. Concurrently, TLR7 and IFN-γ signaling drive STAT1 phosphorylation, which upregulates the transcription factor T-bet and further enhances TLR7 expression, establishing a feed-forward loop (197, 203, 204). IFN-γ and IL-21 produced by follicular or peripheral T helper cells further promote ABC differentiation, expansion, and the generation of pathogenic autoantibodies (205, 206). Indeed, NCF1 R90H mice exhibit a markedly increased proportion of splenic Th1 cells, accompanied by ABC expansion and elevated autoantibody titers in TLR7 agonist–induced lupus (152). In addition, skewing of IgG subclasses toward IgG2 (IgG2c in C57BL/6 mice and IgG2a in BALB/c mice) has been observed in patients with CGD and in *Cybb*^−/−^ mice, correlating with enhanced IFN-γ production under NOX2-deficient conditions (166). IFN-γ, TLR7 signaling and T-bet activation have been shown to promote class-switch recombination toward the IgG2 isotype (201, 207–211). IgG2 isotypes exhibit high-affinity binding to activating Fcγ receptors and strong complement activation, contributing to severe complement-mediated glomerulonephritis in lupus models dominated by IgG2 anti-dsDNA responses (212, 213). Collectively, NOX2 deficiency promotes autoreactive B cell differentiation by enhancing endosomal TLR7/9 signaling in B cells and by creating a Th1-skewed extrinsic environment in which IFN-γ drives ABC expansion, pathogenic IgG2 class switching, and autoantibody-mediated autoimmunity (**Figure 3**).

The enhanced Th1 signaling in NOX2 deficiency triggers inflammatory M1 macrophage and autoreactive B cell differentiation. The IFN-γ therapy is commonly used for CGD patients which prevent serious infections; however, the potential adverse effects and long-term safety remain controversial (168). Although several studies have shown that IFN-γ treatment can partially compensate for the impaired respiratory burst in NOX2 deficiency by enhancing oxygen-independent microbicidal functions in neutrophils and by rewiring monocyte transcriptional programs to upregulate antimicrobial genes, these analyses lack long-term monitoring of treatment effects and immune profile analysis (214–216). Moreover, given the complex immunomodulatory functions of IFN-γ, recombinant IFN-γ therapy should be applied with caution in specific subgroups, particularly patients with CGD who exhibit autoimmune phenotypes and elevated type I interferon-related gene expression, as such treatment may potentially exacerbate autoinflammation and autoimmune pathology (215, 217). These findings highlight the potential risks of conventional IFN-γ therapy and emphasize the need to elucidate cell type-specific mechanisms and treatment responses across different NOX2 subunit variants to enable personalized and safer therapeutic strategies.

## 5 From innate to acquired responses: IL-1β orchestrates the type 1 to type 3 inflammation transition in CGD

CGD is classically defined by an inability to clear pathogens; however, clinical studies reveal that the disease is frequently dominated by sterile hyperinflammation and autoimmunity (99, 146, 218). This defect promotes a pathogenic Th17-type response, centrally mediated by upregulated IL-1β production. The absence of NOX2 signaling triggers NLRP3 inflammasome activity and reshapes the innate-adaptive immune crosstalk, establishing a self-amplifying inflammatory loop. Here, we elaborate on the mechanisms through which IL-1β orchestrates this sustained Th17 hyperinflammation in CGD.

### 5.1 The type 1 to type 3 inflammation transition in different immune cells

Accumulating evidence from murine models and human cohorts indicates that the immunodysregulation of CGD is driven by an excessive Th17-type inflammatory response, characterized by marked expansion of Th17 cells and a concomitant deficiency in Treg function (219, 220). Here, IL-1β acts as the central conductor orchestrating this pathology across both innate and adaptive compartments.

Macrophages are profoundly impacted by NOX2 deficiency, which alters their plasticity and predisposes them to an inflammatory M1 phenotype that orchestrates a pathogenic Th17 response (221). Unlike wild-type macrophages, which rapidly enlarge and mature into a Ly6C^low/-^MHCII^+^CD206^+^CD36^+^ phenotype, CGD macrophages remain physically small and arrested in a pro-inflammatory Ly6C^+^MHCII^–^ state (222). This persistent, immature state predisposes macrophages to intrinsic dysregulation. Furthermore, the loss of NOX2-derived ROS disrupts the activation of Nrf2, a redox-sensitive anti-inflammatory regulator, which fails to suppress inflammatory cytokine production in CGD phagocytes (223, 224).

Concurrently, IL-1β orchestrates the rapid mobilization of neutrophils to the sites of infection by inducing the expression of CXCL8 on endothelial cells, fibroblasts, and keratinocytes as well as by Th17 cells (225–228). This process is further augmented when the secreted IL-17 stimulates epithelial cells to produce a cascade of pro-inflammatory mediators, driving sustained neutrophil activation (229). Moreover, IL-1β acts directly on neutrophils to provide critical anti-apoptotic signals, thereby prolonging their lifespan and establishing a potent self-amplifying feedback loop driven by continuous granulopoiesis (123, 230, 231).

ILCs undergo profound reprogramming within this NOX2-deficient inflammatory milieu, where NLRP3-dependent IL-1β activates NF- κB and STAT3 to drive ROR γt induction, thereby converting T-bet^+^ ILC1s into ROR γt^+^ ILC3s (232–234).Blockade of IL-1 signaling effectively reduces IL-17A-producing ILC3s and ameliorates pathology, supporting a causal role for IL-1 signaling in enforcing a type 1 to type 3 shift (41). This IL-1β-driven plasticity also extends to the adaptive immune system. IL-1β induces hypoxia-inducible factor (HIF-1α) to inhibit differentiation of FOXP3^+^ Tregs and drives their conversion into IL-17-producing Th17 cells (235–237). This highlights a conserved role for IL-1β in enforcing type 3 immunity across both innate and adaptive lineages.

Intrinsic defects in NOX2-deficient B cells further amplify the Th1 to Th17 transition, establishing a reciprocal feed-forward loop with T cells. It has been shown that NOX2 deficiency leads to the upregulation of TLR7 and TLR9 mRNA in B cells, resulting in a hyper-responsive phenotype upon challenge with single-stranded RNA and unmethylated DNA. Crucially, this hyper-activation of B cells results in the excessive secretion of pro-inflammatory cytokines including IL-1β and IL-6 (195, 238). This B cell-derived IL-6 acts directly on naive CD4^+^ T cells to promote STAT3-dependent RORγt expression, thereby driving the initial stages of Th17 differentiation (239). Ultimately, IL-1β functions synergistically with IL-6 and IL-23 not only to amplify the production of effector cytokines including IL-17A, IL-17F, IL-21, and IL-22 but also to induce the expression of the IL-23 receptor on Th17 cells, which is critical for the subsequent survival, proliferation, and pathogenic maintenance of the Th17 population (239–241). In addition, high IL-1 production may stimulate B cells in an autocrine manner to combine with CD40 ligation, enhancing humoral immunity by driving cell expansion and terminal differentiation into antibody-secreting plasma cells (187, 242).

**Figure 4:**
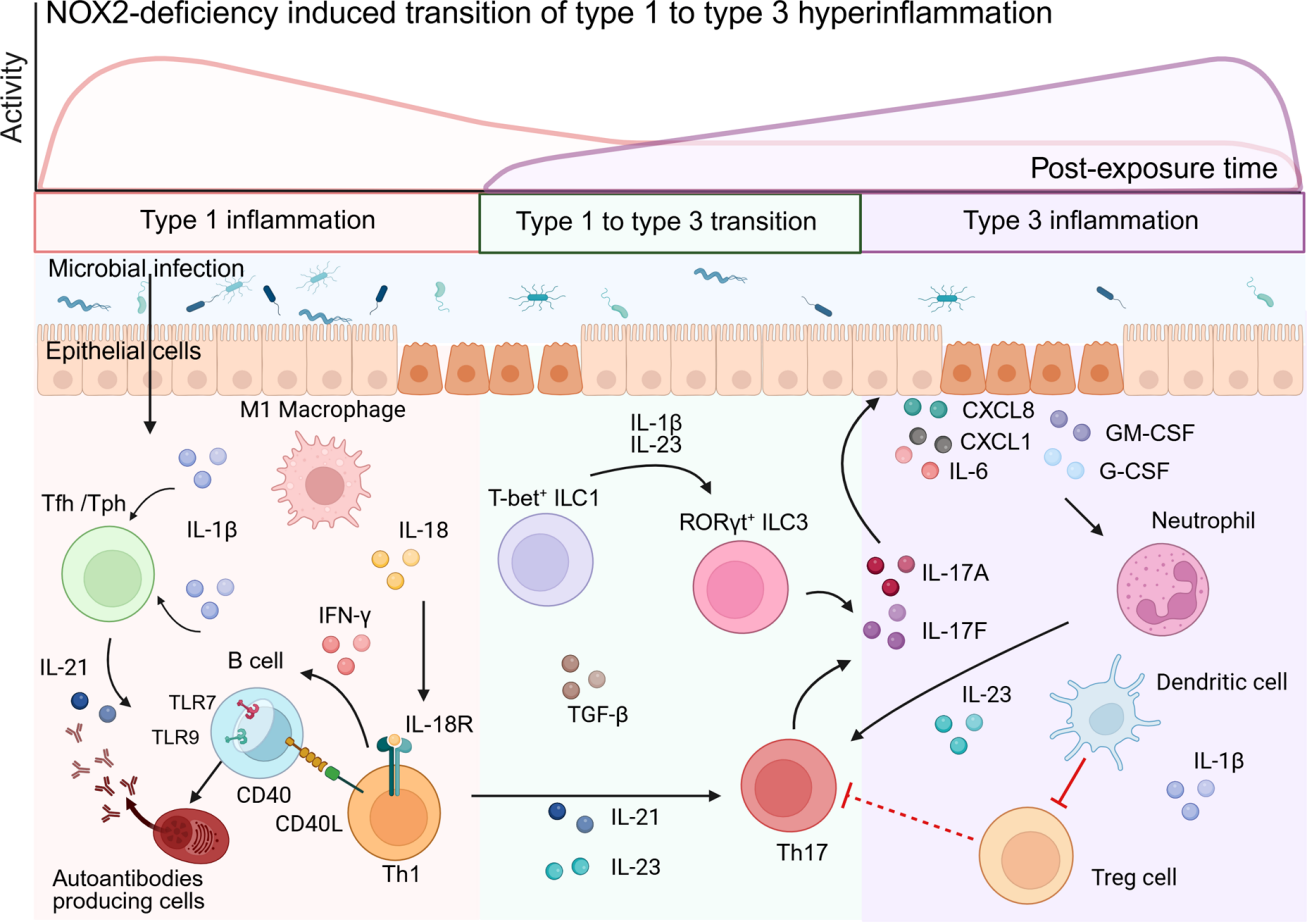
The IL-1β-mediated innate-adaptive immune axis drives the transition from Th1- to Th17-type hyperinflammation in CGD. In the context of NOX2 deficiency, microbial exposure triggers not only Type 1 inflammation but also facilitates a pathogenic cellular transition that amplifies Type 3 inflammation. This results in the coexistence of Th1 and Th17 inflammatory responses characteristic of CGD pathology. M1-type macrophages secrete pro-inflammatory cytokines, including IL-1β, IL-18, and IL-23, in response to microbial infection. Macrophage-derived IL-1β amplifies inflammatory circuits by acting on B and T cells and promoting autoantibody-secreting cell differentiation. Increased B cell-derived IL-1β further enhances Tfh differentiation, leading to IL-21-mediated support of autoantibody production. In parallel, IL-18-driven Th1 polarization and IFN-γ production, along with IL-21, promote autoantibody-secreting cell differentiation in TLR7/9-hyperactivated NOX2-deficient B cells. With IL-1β and IL-23 in the inflammatory milieu, T-bet^+^ ILC1s differentiate into RORγt^+^ ILC3s to generate IL-17A and IL-17F. The IL-17 cytokines act on epithelial cells to produce a series of cytokines and chemokines including IL-6, CXCL8, CXCL1, GM-CSF and G-CSF to recruit and activate neutrophils. Neutrophil-derived IL-23 maintains Th17 cell differentiation. The NOX2-deficient dendritic cells produce IL-1β yet exhibit decreased IL-10 production, dampening Foxp3^+^ Treg cell expansion followed by imbalanced Th17 differentiation.

The convergence of unrestrained innate effector activation and collapsed adaptive tolerance ultimately sustains the severe Th17 hyperinflammation characteristic of CGD patients (**Figure 4**).

## 6 Inflammatory phenotypes in CGD carriers

CGD carriers and individuals with reduced ROS production represent an underrecognized population with significant unmet clinical needs and frequently suffer from recurrent infections and a high prevalence of autoimmune and inflammatory manifestations (16, 107, 146, 243–246). Disease severity in these individuals may be influenced by the degree of X-chromosome inactivation, which determines residual NOX2 activity (16, 247, 248). Proposed pathogenic mechanisms, as observed in CGD patients, include impaired clearance of pathogens and cellular debris, exaggerated inflammatory signaling, and pro-inflammatory immune-cell skewing, as discussed in Section 2. Importantly, aberrant host-microbiota interactions and additional genetic risk variants may further drive autoinflammation and autoimmunity (149, 249, 250).

### 6.1 The immune profiles of CGD carriers

CGD carriers are increasingly recognized to develop infections, autoinflammation, and autoimmune manifestations. Unlike patients with CGD, X-linked CGD (XL-CGD) carriers retain partial NOX2 activity characterized by bimodal ROS production and preserved cytochrome b_558_ expression, which generally protects against severe infections observed in XL-CGD (244). However, many carriers develop recurrent infections, gastrointestinal symptoms, and autoimmune or inflammatory manifestations, including IBD-like colitis, lupus-like disease, alopecia, cytopenias, arthritis, and cardiovascular complications (16, 107, 146, 243–246). Consistent with this inflammatory predisposition, female XL-CGD carriers display elevated type I interferon signatures compared with healthy individuals (163). Supporting these clinical observations, lupus-prone NOX2-deficient models show that heterozygous female mice develop more severe renal pathology, splenomegaly, and increased autoantibody production (155).

X-chromosome inactivation (XCI) is a key determinant of disease variability among female CGD carriers. This epigenetic mechanism balances X-linked gene dosage through XIST-mediated silencing of one X chromosome (251–254). Age-related skewing toward the mutant *CYBB* allele can progressively reduce the proportion of ROS-producing neutrophils, occasionally lowering DHR^+^ cells below 20% and predisposing carriers to infections later in life (16, 247, 248). Clinically, such skewing has been associated with late-onset fungal infections, granulomatous disease, and autoimmune conditions including SLE, DLE, and Raynaud’s phenomenon (135, 247, 248, 255–260). More broadly, dysregulated XCI has also been linked to female-biased autoimmunity, X-linked disorders, and cardiovascular disease (16, 261–265). Supporting this mechanism, PMA-stimulated dihydrorhodamine (DHR) assays show that low ROS production strongly correlates with infection risk but not with autoimmune manifestations, suggesting a greater contribution of immune dysregulation than absolute ROS levels (16).

Several factors may further amplify disease progression in carriers with skewed XCI. First, Increased expression of XCI-escape genes such as *TLR7*, *TASL*, and *CXCR3* may enhance inflammatory signaling (261, 266–270). Consistently, hyperactivation of TLR7 signaling has been observed in B cells and macrophages from CGD patients and NOX2-deficient mice (195, 196). Second, clonal hematopoiesis (CH) may also contribute to disease progression, as next-generation sequencing (NGS) has identified mutations in CH-associated genes such as *DNMT3A* and *ASXL1* in late-onset XL-CGD carriers (271). Expansion of these clones has been linked to increased risks of malignancy, cardiovascular disease, and autoimmune disorders (272). Meanwhile, age-related skewing of XCI has been associated with atherosclerotic cardiovascular disease and myeloid lineage bias, reflected by elevated monocyte-to-lymphocyte ratios and increased monocyte abundance (273). Finally, age-related inflammatory microenvironments may further exacerbate disease through epigenetic alterations and accumulation of senescence-associated secretory phenotype (SASP)-expressing cells that sustain chronic inflammation (56, 274–276).

Autosomal recessive CGD (AR-CGD) is caused by homozygous mutations in *CYBA, NCF1, NCF2*, or *NCF4* and is often associated with consanguinity and higher survival rates (277–280). In contrast to XL-CGD carriers, most heterozygous AR-CGD carriers remain clinically asymptomatic because one functional allele maintains sufficient NADPH oxidase activity. Their DHR assays typically lack the bimodal neutrophil pattern observed in XL-CGD carriers (277, 278, 281, 282).

Recognition of CGD carriers as a clinically relevant population has increased substantially in recent years. Multicenter studies and health-related quality-of-life (HRQL) analyses reveal a significant burden of infections, inflammatory complications, and autoimmune manifestations in this group (146, 243–246, 283, 284). However, major knowledge gaps remain regarding mechanisms of disease onset, longitudinal immune changes, and optimal strategies for monitoring asymptomatic carriers. Improved genetic counseling, family screening, and pregnancy risk assessment will be essential for earlier diagnosis and personalized management (285).

### 6.2 Potential mechanisms underlying “low ROS” phenotypes and inflammation

While excessive ROS production is widely associated with inflammation and tissue damage, insufficient ROS can also drive chronic inflammation and autoimmunity (6, 9, 14, 286, 287). Consistent with the central role of NOX2-derived ROS in immune modulation, reduced NOX2 activity disrupts immune homeostasis and predisposes to autoinflammatory and autoimmune diseases. Such phenotypes are observed in hypomorphic CGD, autosomal recessive CGD, carriers of XL-CGD, pharmacologic NOX2 inhibition, and lupus patients with intermediate ROS production (16, 243, 245, 288).

Genetic studies further support the pathogenic role of reduced ROS signaling. Partial loss-of-function variants in NOX2 subunits that reduce ROS production increase susceptibility to autoinflammatory and autoimmune diseases. For example, hypomorphic *CYBB* mutations have been reported in patients with IBD exhibiting elevated anti-Saccharomyces cerevisiae antibodies (267, 288). Similarly, polymorphisms in *NCF1* and *NCF2* are associated with increased risk of autoimmune diseases including SLE, RA, and MS (146–150, 152, 289). The homozygous *NCF1* R90H mutation causes early-onset interferonopathy with reduced oxidative burst, impaired macrophage efferocytosis, elevated type I interferon signatures, increased Tfh2/Tfr ratios, and enhanced autoantibody production (148, 289). In lupus models, this variant also promotes plasmacytoid dendritic cell activation and expansion of Th1, Th17, and ABC populations (152). Mechanistically, *NCF1* R90H mutation disrupts phagosomal and endosomal maturation by impairing Hv1-dependent phagosomal acidification and *NCF1* endosomal localization (148, 152). Additional hypomorphic variants, including *NCF1*-339 0C genotype and the lupus-associated *NCF2* R395W mutation, similarly reduce ROS production and increase susceptibility to autoimmune inflammation (149, 249). *NCF2* haploinsufficiency further exacerbates lupus development and promotes autoreactive lymphocyte activation (290). These findings highlight the importance of understanding low-ROS states caused by NOX2-related genetic variants, which may facilitate earlier diagnosis and more personalized therapeutic strategies.

Beyond systemic immune dysregulation, NOX2-derived ROS are essential for maintaining immune tolerance to commensal microbes at mucosal interfaces. In NOX2 deficiency, impaired oxidative killing disrupts host-microbe equilibrium, leading to dysbiosis and mucosal immune dysfunction (291–295). Clinically, 40-50% of CGD patients develop IBD-like manifestations increased susceptibility to enteric pathogens including *Enterobacter*, *Enterococcus*, *Escherichia*, and *Klebsiella* species (17, 296–298). Microbiota analyses reveal reduced diversity, fewer short-chain fatty acids (SCFAs)-producing bacteria, and diminished tryptophan-derived metabolites linked to immune dysregulation (299, 300). Reduced SCFAs and AhR ligands may impair IL-22-mediated barrier protection and disrupt the Treg-Th17 balance, favoring Th1 and Th17 expansion (299). Consistently, CGD mice exhibit exaggerated inflammatory responses in colitis models, including increased IL-6, TNF-α, IFN-γ, IL-17A, and NLRP3 signaling along with enhanced T- and B-cell infiltration (301–303). Microbiota-derived factors further promote IFN-γ^+^ Th17 differentiation and pathogenic IgG responses, linking microbial imbalance to autoreactive B-cell activation and systemic inflammation (301–304). In addition, NOX2-derived ROS contribute to early-life host-microbiota equilibrium, shaping macrophage differentiation and colitis susceptibility (250, 305). Overall, these findings indicate that disrupted host-microbiota interactions under low-ROS conditions amplify mucosal inflammation and promote systemic autoimmunity. autoimmunity.

## 7 Conclusion and implications for NOX2-targeting therapies

Given the complex and double-edged sword nature of NOX2-related diseases, therapeutic strategies targeting NOX2 require careful consideration of immune homeostasis. To achieve beneficial NOX2 activation, it is essential to employ a strategy that targets specific immune cell types without eliciting leukocyte inflammatory responses. This approach facilitates the restoration of immune function and promotes tissue immune homeostasis. Beyond pharmacological modulation of ROS, consideration of genetic, metabolic, and microbiome profiles is essential in designing therapeutic strategies that support individualized restoration of immune function.

Small-molecule and RNA interference (RNAi) therapies targeting NOX2 have been developed to be applied in clinical conditions including neurodegenerative and cardiovascular diseases (1, 306–308). However, concerns exist regarding both infection and autoinflammation (110, 309). In contrast, therapeutic approaches aimed at enhancing NOX2-derived ROS production to suppress autoinflammation and autoimmunity remain poorly developed, and no well-established clinical strategies are currently available (310). A better understanding of the link between diminished NOX2-derived ROS production and the development of autoimmunity or autoinflammation not only can help prevent complications in patients with CGD and gene carriers but also benefit individuals with genetic variants associated with reduced ROS production.

Several approaches have been proposed to enhance NOX2-derived ROS. Because cell-type-specific NOX2 activity contributes to diverse disease processes, including infection, autoinflammation, and autoimmunity, selective modulation of NOX2-derived ROS in specific cell populations is particularly important (8). Strategies aimed at enhancing neutrophil-specific NOX2-derived ROS production are currently under development (311, 312). In our previous study, we demonstrated that restoration of impaired gp91^phox^ trafficking using ER-targeted nanoparticles rescued NOX2-derived ROS production and protected neutrophils from mitochondrial damage and apoptosis, highlighting the therapeutic potential of correcting NOX2 dysfunction in CGD (313). This strategy may offer a cell-type-selective approach to enhance NOX2 activity in cells harboring ER-retained immature gp91^phox^. However, approaches targeting NOX2 activity in macrophages or B cells, which also contribute to autoinflammatory and autoimmune responses, have not yet been established.

In summary, this review highlights the critical role of immune regulation in NOX2 deficiency and low-ROS conditions. We summarize the clinical consequences of impaired NOX2 function and pinpoint the key mechanisms by which NOX2-derived ROS reshape innate and adaptive immunity, leading to a broad spectrum of inflammatory and autoimmune disorders. Further mechanistic studies and the rational design of redox-targeted therapies are required to achieve safe and effective clinical interventions and improve long-term outcomes in CGD patients and gene carriers.

## Glossary

13-HODE: 13-hydroxyoctadecadienoic acid
15-HETE: 15-hydroxyeicosatetraenoic acid
ABC: age-associated B cell
AC: apoptotic cell
AD: Alzheimer’s disease
AhR: aryl hydrocarbon receptor
ANCA: anti-neutrophil cytoplasmic antibody
AR-CGD: autosomal recessive chronic granulomatous disease
BBB: blood-brain barrier
BMDC: bone marrow-derived dendritic cell
CGD: chronic granulomatous disease
CH: clonal hematopoiesis
CIA: collagen-induced arthritis
CII: collagen type II
CPM: counts per million
CTL: cytotoxic T lymphocyte
DAMP: damage-associated molecular pattern
DLE: discoid lupus erythematosus
ESR: erythrocyte sedimentation rate G-CSF, granulocyte colony-stimulating factor
GILT: IFN-γ-inducible lysosomal thiol reductase
GPI: glucose-6-phosphate isomerase
GWAS: genome-wide association studies
HEL: hen egg white lysozyme
HRQL: health-related quality of life
IBD: inflammatory bowel disease
iDC: immature dendritic cell
IDO: indoleamine 2,3-dioxygenase
IEI: inborn error of immunity
IFN: interferon
ILC: innate lymphoid cell
iNKT: invariant natural killer T cell
LAP: LC3-associated phagocytosis
LO: 12/15-lipoxygenase
LTB₄: leukotriene B4
MODS: multiple organ dysfunction syndrome
MOG: myelin oligodendrocyte glycoprotein
MS: multiple sclerosis
mCAIA: mannan-enhanced type II collagen antibody-induced arthritis
NADPH: nicotinamide adenine dinucleotide phosphate
NET: neutrophil extracellular trap
NK: natural killer cell
NOX2: NADPH oxidase 2
NOXs: NADPH oxidases
OVA: ovalbumin
PAMPs: pathogen-associated molecular patterns
PR3: proteinase 3
PtdSer: phosphatidylserine
PTPs: protein tyrosine phosphatases
PVOD: pulmonary veno-occlusive disease
RA: rheumatoid arthritis
ROS: reactive oxygen species
SCFA: short-chain fatty acid
SIRS: systemic inflammatory response syndrome
SLE: systemic lupus erythematosus
SYK: spleen tyrosine kinase
Tfh: T follicular helper cell
TLR: Toll-like receptor
Tph: peripheral helper T cell
Treg: regulatory T cell
XCI: X-chromosome inactivation
XL-CGD: X-linked chronic granulomatous disease
ZIA: zymosan-induced arthritis.
